# Gun Violence Exposure and Quality of Life in Nine US States

**DOI:** 10.1007/s11524-024-00891-7

**Published:** 2024-07-29

**Authors:** Jennifer Paruk, Daniel Semenza

**Affiliations:** 1 New Jersey Gun Violence Research Center, Rutgers University, Piscataway, New Jersey 08854 USA; 2 Department of Urban-Global Public Health, Rutgers University, Piscataway, New Jersey 08854 USA; 3 Department of Sociology, Anthropology, and Criminal Justice, Rutgers University, Camden, NJ USA

**Keywords:** Gun violence, Quality of life, Community health

## Abstract

Direct and indirect gun violence exposure (GVE) is associated with a broad range of detrimental health effects. However, much of this research has examined the effects of a single type of GVE (e.g., being shot) on discrete outcomes (e.g., daily pain, PTSD). Since people may experience numerous types of GVE (e.g., being threatened with a gun and hearing gunshots in their neighborhood) with broad effects on their well-being, we study the association between four types of direct and indirect GVE and five aspects of quality of life (overall, physical, psychological, social, and environmental). Using a representative sample of adults from nine states (*N* = 7455), we find that witnessing/hearing about a shooting in one’s neighborhood was the most commonly experienced GVE associated with significant decreases in all five types of quality of life. Cumulative GVE was also associated with significant decreases in overall physical, psychological, social, and environmental quality of life. For example, individuals with four GVEs had an adjusted average physical quality of life that was 11.14 points lower and environmental quality of life that was 7.18 points lower than individuals with no GVE. Decreasing gun violence is a critical component of improving community health and well-being.

In 2021, more than 20,000 people died from firearm homicide [1]. Survivors of shootings or other forms of gun violence exposure (GVE) experience adverse effects on their physical and mental health, as well as daily functioning. Hospitals report over 34,000 emergency room visits per year due to non-fatal firearm assaults [2], and these firearm violence survivors require substantial follow-up health care [3], suffer functional disabilities [4], have problems with mobility [5], and experience daily pain as a result of their injury [6]. Surviving a shooting is also associated with an increased number of subsequent mental health visits [3], a greater number of bad mental health days in the past month [7], an increase in psychiatric disorders and substance use disorders [3], and screening positive for PTSD [6]. Furthermore, survivors report problems sleeping, irritability, and hypervigilance regarding personal safety. [8]

Experiencing other types of direct GVE (e.g., being shot at but not hit or being threatened with a firearm) and indirect GVE (e.g., having a friend or family member shot or secondary survival) are also associated with adverse health effects. Those threatened with firearm violence report experiencing severe distress [9] and fear after the incident [10], poorer self-rated health and significantly more bad mental health days [7], suicidal ideation [11], and problems sleeping [12]. Witnessing a shooting or knowing someone who has been shot is associated with psychological distress, depression, and other mental health diagnoses,[13–15] parent aggravation [13], lower scores of self-rated health [7], and problems sleeping [11]. Those caring for loved ones who had been shot also report experiencing vicarious trauma [16].

Despite research on poor health associated with specific forms of direct and indirect GVE, there remains limited research on the health effects of cumulative GVE (e.g., both being threatened with a firearm and knowing someone who died in a shooting). However, some studies suggest that experiencing more than one form of GVE is associated with poorer mental and physical health outcomes [11, 12]. In nationally representative samples of Black adults and American Indian and Alaska Native adults, the more GVE forms individuals experienced, the greater their risk of having bad physical health days within the past month, indicating a dose-response relationship [7]. Another study of Black Americans found that experiencing three or more types of GVE was associated with greater functional disability, particularly among women [17]. Most quantitative studies on the effects of individual or cumulative GVE have focused on discrete outcomes such as pain [3] or depression, [13] which likely does not capture the full range of harms to quality of life experienced by those exposed to gun violence.

As such, it is critical to consider the association between GVE and quality of life to provide greater insight into how GVE shapes overall well-being. Quality of life entails one’s perception of their position in life based on their physical and mental health, social relationships, and environment [18]. Quality of life emphasizes health beyond considerations of morbidity and mortality, underscoring comprehensive well-being as a fundamental objective in health care [19]. Lower quality of life has been associated with direct violence victimization, including intimate partner violence [20] and sexual trauma [21]. To our knowledge, only one study has analyzed the association between GVE and quality of life. Herrera-Escobar and colleagues [6] found that firearm injury survivors had significantly worse physical and mental health-related quality of life than matched survivors who experienced motor vehicle crashes in Boston. However, this study only examined direct survivors of shootings with non-fatal injuries (no other types of GVE such as secondary survival or broader community exposure) and therefore could not examine the effects of multiple gun violence exposures. In the present study, we examine how four types of GVE and cumulative GVE are associated with quality of life.

## Data and Methods

### Data

We gathered data from non-institutionalized adults living in nine states (MS, NJ, CO, TX, MN, WA, PA, OH, and FL) with Ipsos KnowledgePanel, the largest probability-based online panel of adults in the USA. States were chosen given their diversity in geodemographics, politics, and rates of firearm injury. Data collection occurred in June and July 2023. Participants were invited to complete surveys via email, with a reminder sent three days later to those who had not yet responded. The median time to complete the survey was 25 min. Out of 13,568 surveys distributed, 8490 (63%) adults opened the survey and responded to the informed consent question, with 7785 (92%) agreeing to take part in the research study. The survey and study received approval from the Rutgers University Institutional Review Board. Design weights for the full sample of all nine states were calculated using an iterative proportional fitting procedure, which adjusted weights to match geodemographic distributions of the state populations according to the 2021 American Community Survey benchmarks for race/ethnicity, gender, education, and household income. These weights were trimmed and normalized to equal the total number of qualified respondents.

### Measures

#### Outcomes

All outcome variables were derived from the World Health Organization’s (WHO) Quality of Life – Brief Scale (WHOQOL-BREF) [22]. The WHO defines the quality of life as “an individual’s perception of their position in life in the context of the culture and value systems in which they live and in relation to their goals, expectations, standards, and concerns.” [23] The WHOQOL-BREF contains a subset of 26 items from the larger 100-item standard WHOQOL instrument covering two general questions and four main domains of quality of life: physical, psychological, social, and environmental. A full list of all WHOQOL-BREF items can be found in the Appendix. This scale has been used extensively as a valid and reliable means of assessing quality of life in diverse settings and research designs [24, 25]. 

All quality-of-life questions asked respondents to consider their answers regarding the past two weeks of their lives. *Physical quality of life* was measured using seven items such as, “To what extent do you feel that (physical) pain prevents you from doing what you need to do?” and “How much do you need any medical treatment to function in your life?” Responses for these items ranged from 1 (not at all) to 5 (an extreme amount) (alpha = .869). Reverse coding was used for two items in the physical health domain to ensure directional scaling consistency. *Psychological quality of life* was measured using five items including, “How much do you enjoy life?” and “How well are you able to concentrate?” Responses for these items ranged from 1 (not at all) to 5 (an extreme amount) (alpha = .856). One item from the original WHOQOL-BREF regarding feelings such as blue mood, despair, anxiety, and depression was unavailable in the survey and omitted from our measure. *Social quality of life* was measured using three items including, “How satisfied are you with your personal relationships?” and “How satisfied are you with the support you get from your friends?” Responses for these items ranged from 1 (very dissatisfied) to 5 (very satisfied) (alpha = .773). Finally, *environmental quality of life* was measured using eight items such as, “How satisfied are you with the condition of your living place?” and “To what extent do you have the opportunity for leisure activities?” Responses for these items ranged from 1 (very dissatisfied) to 5 (very satisfied) (alpha = .773). We measured *overall quality of life* using the single item, “How would you rate your quality of life?” Responses ranged from 1 (very poor) to 5 (very good).

#### Gun violence exposures

We measured gun violence exposure types using four binary items (0 = no; 1 = yes). *Threatened with a firearm* was measured with the question, “Have you ever been threatened with a firearm by another person?” *Shot with a firearm* was measured using the question, “Have you ever been shot on purpose by another person with a firearm?” We measured *family/friend shot* with the question, “Do you personally know someone, such as a friend or family member, who has been shot on purpose by another person with a firearm?” Finally, we measured *witnessed/heard* about a shooting using the question, “Have you ever witnessed or heard about someone being shot intentionally by another person with a firearm in your neighborhood?” We measured *cumulative gun violence exposure* by adding all four firearm violence exposure items to create a scale ranging from 0 to 4.

#### Controls

Control measures include *sex* (male/female), *age* (18–29, 30–44, 45–59, 60+), *education* (no high school degree, high school degree, some college, or bachelors or more), *race/ethnic identity* (Black, Hispanic, White, Other/Multi-racial), *household income* (<$25,000, $25,000 to $74,999, $75,000 to $149,999, $150,000+), *employment status* (working full time, working part-time, not working), *marital status* (married, widowed, divorced/separated, never married), *home ownership status* (own/rent), and *state*. We also controlled for the *metropolitan area* (yes/no) using the Metropolitan Statistical Area (MSA) designation and *perceived neighborhood safety.* Perceived neighborhood safety was measured using nine items such as “I do not feel safe being home alone” and “I am afraid of somebody breaking into my home and stealing or damaging things,” with response options that ranged from strongly disagree (1) to strongly agree (7) (alpha = 0.94). Items were reverse coded and summed for a final score so that higher scores indicate higher perceived neighborhood safety. We also controlled for lifetime *physical abuse*, *emotional abuse*, and *sexual abuse* from a loved one or someone with whom the participant was very close (yes/no), using three items from the Brief Betrayal Trauma Survey [26]. 

### Analytic Strategy

Scoring for quality-of-life measures followed the WHO-recommended transformations and scoring for the WHOQOL-BREF [27]. All five outcome measures were transformed to range from 0 to 100 to aid in interpretation and comparison across measures. Raw scores were summed for each of the four quality-of-life domain outcomes and then transformed using the following equation:

$$\text{Transformed Scale}=\left[\frac{\left(\text{Actual raw score}-\text{lowest possible raw score}\right)}{\text{Possible raw score range}}\right]\times 100$$

where “Actual raw score” is the value achieved through summation, “lowest possible raw score” is the lowest value that could occur through summation (4 for all items), and “possible raw score range” is the difference between the maximum and minimum possible raw scores. The single overall quality of life item was recoded from 1 to 5 on the original scale to range from 0 to 100.

We calculated descriptive statistics and then used ordinary least squares (OLS) models to regress all quality-of-life outcome measures on the main exposure variables and all controls. Separate models with full controls were run to assess associations for individual GVE types and cumulative exposure with each quality-of-life outcome. After conducting multivariate linear regressions with cumulative GVE, we calculated the adjusted means for each type of quality of life. Listwise deletion was used to account for missing data in the models (5% missing). All analyses were conducted in Stata 18 using the *svy* commands for weighted data.

## Results

Table 1 includes descriptive statistics of GVEs. Of the four types of GVE, witnessing or hearing about a shooting in the neighborhood was the most common (22%), followed by knowing someone close to them (family or friend) who was shot (19%), being threatened with a gun (13%), and shot with a gun (2%). In total, 37% of participants experienced at least one type of GVE. Cumulative GVE ranged from 0 to 4, with an average of 0.55.

**Table 1:** Weighted Descriptive Statistics (*N* = 7785)

	*N* (%)		*N* (%)
Quality of life: mean (SD)		Marital status	
Overall	78.89 (0.37)	Married	4343 (56%)
Physical	74.55 (0.32)	Widowed	321 (4%)
Psychological	69.94 (0.35)	Divorced/separated	957 (12%)
Social	68.13 (0.38)	Never married	2164 (28%)
Environmental	75.60 (0.32)	Past victimization	
Gun violence exposures (GVE)		Physical abuse	1098 (14%)
Threatened with gun	970 (13%)	Sexual abuse	1162 (15%)
Shot with gun	117 (2%)	Emotional abuse	2502 (32%)
Family/friend shot	1468 (19%)	Metropolitan area	6,952 (89%)
Witnessed/heard shooting	1675 (22%)	Homeownership status	
Cumulative GVE: Mean (SD)	0.55 (0.83)	Own	6002 (77%)
Female	4062 (52%)	Rent	1783 (23%)
Age		State	
18–29	1304 (17%)	NJ	657 (8%)
30–44	2086 (27%)	PA	957 (12%)
45–59	1952 (25%)	OH	868 (11%)
60+	2443 (31%)	MN	417 (5%)
Education		FL	1651 (21%)
No HS	408 (5%)	MS	205 (3%)
HS degree	2412 (31%)	TX	2027 (26%)
Some college	2291 (29%)	CO	429 (6%)
Bachelors or more	2674 (34%)	WA	572 (7%)
Race/ethnicity			
White	4764 (61%)		
Black	825 (11%)		
Hispanic	1535 (20%)		
Other/2+	660 (8%)		
Household income			
<$24,999	2240 (29%)		
$25,000 to $74,999	1311 (17%)		
$75,000 to $149,999	2594 (33%)		
$150,000+	1640 (21%)		
Employment status			
Working full time	3790 (49%)		
Working part-time	1116 (14%)		
Not working	2879 (37%)		
Neighborhood safety: mean (SD)	49.77 (0.20)		

Associations between individual GVE types and all quality-of-life outcomes are depicted in Table 2. Witnessing or hearing shootings in the community was associated with a 2.33% reduction in overall quality of life. All GVE types were associated with a reduction in physical quality of life, ranging from 2.49% (family or friend shot) to 6.98% (being shot with a gun). Witnessing or hearing shootings in the community was associated with a reduction in psychological (1.92%) and social quality of life (2.98%). Both being threatened with a gun (1.99%) and witnessing or hearing about shootings in the community (3%) were associated with reductions in environmental quality of life.

**Table 2:** Gun violence exposures and quality of life

	Coef.	S.E.	95% CI
	**Overall**		
Threatened with gun	−0.29	1.15	−2.54, 1.97
Shot with gun	−3.52	3.30	−9.99, 2.96
Family/friend shot	−1.13	1.01	−3.11, 0.85
Witnessed/heard shooting	−2.33*	1.00	−4.30, −0.36
Cumulative	−1.29**	0.49	−2.26, −0.32
*R*^2^		0.19	
	**Physical**		
Threatened with gun	−2.59**	0.86	−4.28, −0.89
Shot with gun	−6.98*	2.71	−12.29, −1.67
Family/friend shot	−2.49**	0.8	−4.07, −0.91
Witnessed/heard shooting	−2.77***	0.73	−4.21, −1.34
Cumulative	−2.78***	0.38	−3.54, −2.03
*R*^2^		0.28	
	**Psychological**		
Threatened with gun	−0.05	0.97	−1.96, 1.85
Shot with gun	0.65	2.59	−4.43, 5.73
Family/friend shot	−0.63	0.94	−2.47, 1.21
Witnessed/heard shooting	−1.92*	0.82	−3.54, −0.31
Cumulative	−0.84*	0.41	−1.64, −0.04
*R*^2^		0.25	
	**Social**		
Threatened with gun	−1.15	1.1	−3.4, 1.06
Shot with gun	5.17	3.7	−2.2, 12.4
Family/friend shot	−0.25	1.1	−2.4, 1.86
Witnessed/heard shooting	−2.98**	0.9	−4.7, −1.23
Cumulative	−0.99*	0.48	−1.93, −0.05
*R*^2^		0.19	
	**Environmental**		
Threatened with gun	−1.99*	0.78	−3.53, −0.45
Shot with gun	−1.89	2.12	−6.04, 2.27
Family/friend shot	−0.80	0.74	−2.25, 0.64
Witnessed/heard shooting	−3.00***	0.63	−4.24, −1.75
Cumulative	−1.80***	0.36	−2.50, −1.09
*R*^2^		0.41	

^***^p < .001

^**^p < .01

^*^p < .05

All models control for prior physical abuse, prior sexual abuse, prior emotional abuse, perceived neighborhood safety, sex, age, education, race, income, marital status, employment status, home ownership status, metropolitan status, and state

**Fig. 1:**
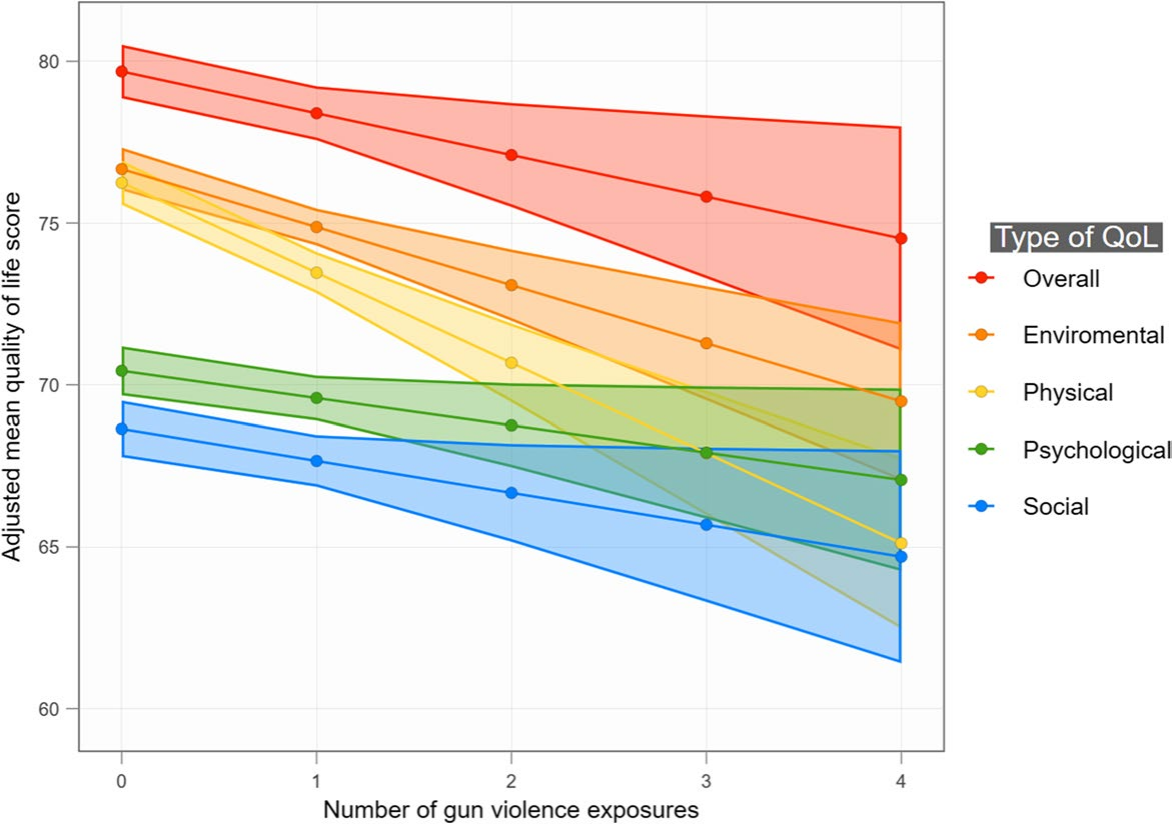
Quality of life (QoL) adjusted means

Table 2 also shows that cumulative GVE was associated with a decrease in all types of quality of life. Each increase in the number of GVE types was associated with a 1.29% decrease in overall quality of life, 2.78% decrease in physical quality of life, 0.84% decrease in psychological quality of life, 0.99% decrease in social quality of life, and 1.80% decrease in environmental quality of life. Using these models, Fig. 1 shows the adjusted means for each type of quality of life. When GVE is increased from 0 to 4, the adjusted mean quality of life decreases 5.17% (from 79.69 to 74.52) in overall quality of life, 11.14% (from 76.25 to 65.11) in physical quality of life, 3.36% (from 70.43 to 67.07) in psychological quality of life, 3.94% (from 68.64 to 64.70) in social quality of life, and 7.18% (from 76.67 to 69.49) in environmental quality of life.

## Discussion

In this representative sample of adults from nine states, our study of the relationship between GVE and quality of life produced two main findings. First, witnessing/hearing about shootings in the neighborhood was the most common GVE and was significantly associated with decreases in all types of quality of life (overall, physical, psychological, social, and environmental). Second, cumulative GVE was also associated with decreases in all types of quality of life, and those with the highest exposure to gun violence reported the lowest adjusted mean quality of life.

Even though only a small portion of participants reported experiencing a direct physical injury from GVE (2%), we found the largest adjusted mean difference between those with zero GVE and four GVE in physical quality of life (11% difference), emphasizing that indirect GVE can still significantly affect physical well-being. This is consistent with research that shows how witnessing violence can become “biologically embedded,” [28] leading to negative physical health outcomes. Exposure to violence is associated with physiological and cellular markers of stress, [29] which affects numerous biological systems related to stress [30]. For example, Goin and colleagues [31] found that pregnant women in neighborhoods with high rates of firearm violence were more likely to have spontaneous preterm births, mediated by infections and substance use, highlighting the impact of chronic gun violence stressors on immune function and pregnancy outcomes. Fear of violence can also affect how residents exercise and move around their neighborhoods, affecting both short- and long-term physical well-being. Both perceptions of neighborhood safety and crime rates are associated with decreased physical activity, [32] which can lead to numerous negative physical [33] and mental health outcomes [34] and decreased quality of life. [35]

These findings highlight that healthcare facilities and gun violence prevention programs could expand services to those indirectly exposed to GVE. Drawing from interviews with Black women taking care of loved ones who had been shot, Aguilar and colleagues [16] suggest that hospitals and other gun violence prevention programs should provide resources to families to reduce vicarious trauma and enable them to better care for their loved ones while also prioritizing their own health and well-being. The caregivers they interviewed specifically suggested that support groups, led by staff who have faced similar challenges and lived experiences, could help caregivers connect with and learn from other caregivers in similar situations. As evidenced in our findings, those with high levels of cumulative GVE report low levels of quality of life in many domains. While deeper structural and long-term efforts are needed to reduce violence to improve broader community well-being, Aguilar and colleagues [16] suggest that these support groups could also be an avenue for hospitals or other programs to provide connections to resources.

This study has several limitations that provide opportunities for future research. First, the data are cross-sectional, precluding causal inferences for the relationship between GVE and quality of life. Second, the findings are only generalizable to the nine states included in the sample. Although these states are diverse in their demographics, politics, and rates of firearm ownership and firearm violence, it is possible that the relationship between GVE and quality of life looks different in other states. Third, although we controlled for numerous covariates that could be associated with GVE and quality of life, it is likely we did not control for all possible covariates. Finally, there was a low prevalence of being shot (2%), which limits precision in the interpretation of the association between being shot and quality of life.

Despite these limitations, this study expands our knowledge of the effects of GVE in meaningful ways. One study previously found an association between being shot with decreased physical and mental quality of life, but by using additional forms of GVE, we find that witnessing/hearing about a shooting in the neighborhood and increases in cumulative GVE are associated with lower quality of life of all types (overall, physical, psychological, social, and environmental). This study thus contributes to a growing body of evidence showing that different forms of direct and indirect GVE, as well as cumulative exposure, are associated with poorer health. Our findings underscore that GVE is harmful beyond discrete measures of mental and physical health, affecting many interrelated aspects of daily living and well-being. Reducing gun violence must be a critical component of any policy effort to support everyday well-being and community thriving.

## Data Availability

We will provide the requester with results and output or with specific deidentified components of the dataset that address the request.

## Appendix. WHOQOL-BREF

**Overall Quality of Life**

1. Thinking about the last two weeks, how would you rate your quality of life?

**Physical Quality of Life**

Thinking about the past two weeks…

1. To what extent do you feel that (physical) pain prevents you from doing what you need to do?
2. Do you have enough energy for everyday life?
3. How well are you able to get around?
4. How satisfied are you with your sleep?
5. How satisfied are you with your ability to perform your daily living activities?
6. How satisfied are you with your capacity to work?
7. How much do you need any medical treatment to function in your daily life?

**Psychological Quality of Life**

Thinking about the past two weeks…

1. How much do you enjoy life?
2. To what extent do you feel your life to be meaningful?
3. How well are you able to concentrate?
4. How satisfied are you with yourself?
5. Are you able to accept your bodily appearance?

**Social Quality of Life**

Thinking about the past two weeks…

1. How satisfied are you with your sex life?
2. How satisfied are you with the support you get from your friends?
3. How satisfied are you with your personal relationships?

**Environmental**

Thinking about the past two weeks…

1. How safe do you feel in your daily life?
2. Have you enough money to meet your needs?
3. How available to you is the information that you need in your day-to-day life?
4. How satisfied are you with your transport?
5. How healthy is your physical environment?
6. To what extent do you have the opportunity for leisure activities?
7. How satisfied are you with the condition of your living place?
8. How satisfied are you with your access to health services?
